# Supplementary material for: Patterns of Intron Gain and Loss in Fungi
Source: PLoS Biol. 2004 Nov 30;2(12):e422. doi: 10.1371/journal.pbio.0020422 (PMC532390; doi:10.1371/journal.pbio.0020422)
Supplement: Table S1 — Also available at http://genes.mit.edu/NielsenEtAl/. (4.3 MB ZIP). [file pbio.0020422.st001.zip › NielsenEtAl/html/1366.html]

AN6293.1.NCU03352.1.MG03261.1.FG01562.1


```
 CLUSTAL W (1.82) Multiple Sequence Alignments - Introns Inserted


Sequence 1: NCU03352.1	470 aa
Sequence 2: FG01562.1	524 aa
Sequence 3: MG03261.1	525 aa
Sequence 4: AN6293.1	556 aa
Alignment Length: 607 aa
Number Identitical Residues: 151 aa
Alignment Score (without introns) 7843


MG03261.1 	------------------------MEPAKGKDASPSDDAAKPDTE-VRHPLKASRSSGTE
NCU03352.1	--------------------MTG-TEATEKPNGKEAGTKDITKSG-SDTKPKDHHPTPAD
FG01562.1 	--------------------MTDRNPATAAKSAKQELAKTDDGKA-VNRDKSSNTATSSN
AN6293.1  	MTEKNTTQPGSAPDQYAGNGTAENTGPSKTLDGNKVGRVDSNAKENAQKSPASKTESTPA
          	 :...::...::... :... :    .:   ...         . .          : . 

MG03261.1 	EQGKAPKKRRKVNHA1CVYCRRS0------~---------~RNIGHLCHDEPREHESKKA
NCU03352.1	DVQKAPKKRRKVNHA1CLYCRRS0------~ERPCTRCIK~RNIGHLCHDEPRDTESRKA
FG01562.1 	QKTTPAKKRRKVNHA1CVYCRRS0HMTCDL0ERPCTRCIK~RNIGHLCHDEPRDADSKKT
AN6293.1  	GTRTSPKKRRKVNHA1CVYCRRS0------~---HMTCDS0RNIGHLCHDEPREP-SKRS
          	   ...********* *:*****              . . ************:  *:::

MG03261.1 	KSALG-------TSADDSEHQTDTTQTSRDTQAAASTMAAYDGRIDAVGTGLDNGAATAV
NCU03352.1	KSVLG------TSTLHDSESQPDIGRNATD-KAMRP--PGFDS---GMGNGSVQVAGAAA
FG01562.1 	KSVQSSQSIQAPSVVDESDAQSDMARSSIS-STMGPPPPTFDT---TRQRGSKSFGG--V
AN6293.1  	RSEHE-------QSAADEEGSSNNEYSKVH--AMPRKVDIQDA------AGQQILADGSL
          	:*              :.: ..:   .     :        *        *    .  : 

MG03261.1 	LGRGNPL-LVQQSPLTGVRPNAMG-SENLNSC1KV~PGFSDGWINQSHFHDMHGYHPSFN
NCU03352.1	VGRGAPLQLVQPGSVAGIQASAL--GGS--MN~QC1LLEESAFMSDD-------------
FG01562.1 	LGQGSPLSIVQPGQVSGLQGNELNNGGSSNAN~QF1VGFQDAWVTAQNHFHDMHSYHPNY
AN6293.1  	GLTPSSMNAVQPGPMSSSTSQNMS---VTSQQ~QL1LGYNDWVGGQNQFQDMHTFHPSYM
          	     .:. ** . ::.   . :.     .   :     ..     .          .  

MG03261.1 	LITPEVTNEFNLLSDFLNGSLLEDGSLLQDEQSQTLPPGTAQADTIVSS-----FLNSNG
NCU03352.1	-------HGLILGANQSAVSGLPN-ANNNGNNASSNNKGNSSSTSGMLP-------PSAT
FG01562.1 	LIAPEVTHEFNLLNDFLHTSLLDDGGVSSEDQQSSAFKRTSQSQSEMLPRFGNNNNPMNA
AN6293.1  	FNAPEVTNEYNLLGDFLSNSLLDDGSIFQNEDMQRMYSDPTLINSMAVLG-----GPSTS
          	  :.. ::   *  :    * * :..  . :: .      :   :               

MG03261.1 	ENSTTMNGTENSMLPPSAIPHQKESLSTSQGELGKSIARPSSTVPIDKARE-YYLQAADP
NCU03352.1	QGT--------SMLPP------------SSD-QTTAIGKPASTNLDNARDA-YYLQAADP
FG01562.1 	GGSNSMTNMSGSMLPP------------PPNKEGKNIPRPGSVVPVDKARE-YYLQAADP
AN6293.1  	LLQQS------QTLQP------------QQSQQNQGDTASGATIGNDKARETYYMTAADP
          	    :      . * *              ..       ..:.   :    :**: ****

MG03261.1 	SGNDTAEERMQRLLKAKYDAGLLKPFNYIKGYSRLSNYLDSHIAATSKQKILRQLDRFRP
NCU03352.1	SGNDTPEERMQRLLRAKYEAGLLKPFNYILGYKRLSDYLDGHVSPTSKQKILKQIDRFRP
FG01562.1 	SGNDTPEERMGRVLRAKYDAGLLKPFNYINGYTRLGTYLDSHIAASSKSKIIKTINSFRP
AN6293.1  	AGSDPPEERMNKLLKAKYDAGLLRPFNYVKGYARLSAYMEKNLQVSSRQKIARQLDKFRP
          	:*.*..**** ::*:***:****:****: ** **. *:: ::  :*:.** : :: ***

MG03261.1 	KFREKIHALTDIELVFVEMWFEKTLMDYDRVFASMAIPACCWRRTGEIFRGNKEMAELIN
NCU03352.1	KFREKIQLLTDMDLLMVEMWFERTLLEYDRVFASMAVPACCWRRTGQIFRGNKEMAELIG
FG01562.1 	KFREKAQALTDMELVYVEMWFEKQLMDYDRVFASMAVPACCWRRTGEIFRGNKEMAELIG
AN6293.1  	KFRDRMQKLTDIELILVEMWFERSLMEYDRVFASMAIPACCWRRTGEIFRGNKEMAELIG
          	***:: : ***::*: ******: *::*********:*********:************.

MG03261.1 	VPVDDLRD0GKIALHEILTEESVVRYWEEFGTIAFDPAHDTLLTACTLKNPDDKSELPAI
NCU03352.1	VPVESLRG0GQIALHEILTEESNVRYWEEFGTIAFDPAHDTLITACSLKNPNDDKGTKVV
FG01562.1 	VSVAQLRD0GKIALHEILTEESMVRYWEEFGTIAFDPAHETLLTACSLKNPSAESTHPIV
AN6293.1  	VPIELLRD0GKLAIHEIIVEDQLVSYWEKFGAIAFDNTQKAMLTSCTLKNPNATSPTEGI
          	*.:  **. *::*:***:.*:. * ***:**:**** ::.:::*:*:****.  .    :

MG03261.1 	KCCFSLKIRRDDHKI2RNNDNDPHGQTLMSGDWIAPYQGNK~LPRHEVL---------~-
NCU03352.1	NCCFSFRIRRDDHKI2A-----------------PVIRNAD~EQCDTCFP--------~-
FG01562.1 	KCCFSFMIRRDEHKL2P-----------------ALIVGNF~LPHDPPTQ--------~-
AN6293.1  	PCCFSFTIRRDNHNI2FLTPLILVRLLLLEISYQRNGIPNR1KKHSSRFQDQTQSLSV1C
          	 ****: ****:*::   .          . .                  ..:.: :  .

MG03261.1 	-----------------
NCU03352.1	-----------------
FG01562.1 	-----------------
AN6293.1  	RGALKQLTRFAIQKLLF
          	 .: .. :  : ..
```
